# FDG-PET Imaging for Hodgkin and Diffuse Large B-Cell Lymphoma—An Updated Overview

**DOI:** 10.3390/cancers12030601

**Published:** 2020-03-05

**Authors:** Conrad-Amadeus Voltin, Jasmin Mettler, Jirka Grosse, Markus Dietlein, Christian Baues, Christine Schmitz, Peter Borchmann, Carsten Kobe, Dirk Hellwig

**Affiliations:** 1 Department of Nuclear Medicine, Faculty of Medicine and University Hospital Cologne, University of Cologne, 50937 Cologne, Germany; jasmin.mettler@uk-koeln.de (J.M.); markus.dietlein@uk-koeln.de (M.D.); carsten.kobe@uk-koeln.de (C.K.); 2 Department of Nuclear Medicine, University Hospital Regensburg, 93053 Regensburg, Germany; jirka.grosse@ukr.de (J.G.); dirk.hellwig@ukr.de (D.H.); 3 Department of Radiation Oncology and Cyberknife Center, Faculty of Medicine and University Hospital Cologne, University of Cologne, 50937 Cologne, Germany; christian.baues@uk-koeln.de; 4 Department of Hematology, West German Cancer Center (WTZ), University Hospital Essen, University of Duisburg-Essen, 45147 Essen, Germany; christine.schmitz@uk-essen.de; 5 Department of Internal Medicine I, Center for Integrated Oncology Aachen-Bonn-Cologne-Dusseldorf (CIO ABCD), Faculty of Medicine and University Hospital Cologne, University of Cologne, 50937 Cologne, Germany; peter.borchmann@uk-koeln.de

**Keywords:** Hodgkin lymphoma, diffuse large B-cell lymphoma, positron emission tomography, staging, response assessment

## Abstract

Since the mid-1990s, ^18^F-fluorodeoxglucose (FDG)-positron emission tomography (PET) in combination with computed tomography has come to play a prominent role in the management of malignant lymphomas. One of the first PET applications in oncology was the detection of lymphoma manifestations at staging, where it has shown high sensitivity. Nowadays, this imaging modality is also used during treatment to evaluate the individual chemosensitivity and adapt further therapy accordingly. If the end-of-treatment PET is negative, irradiation in advanced-stage Hodgkin lymphoma patients can be safely omitted after highly effective chemotherapy. Thus far, lymphoma response assessment has mainly been performed using visual criteria, such as the Deauville five-point scale, which became the international standard in 2014. However, novel measures such as metabolic tumor volume or total lesion glycolysis have recently been recognized by several working groups and may further increase the diagnostic and prognostic value of FDG-PET in the future.

## 1. Introduction

Hodgkin lymphoma is a hematologic malignancy with one of the best long-term outcomes after first-line treatment. Ongoing efforts to improve therapy effectiveness have resulted in a 5-year relative survival rate of about 90% for patients diagnosed between the ages of 20 and 64 years [1]. Treatment is selected depending on individual factors and consists of chemotherapy alone or chemotherapy followed by irradiation [2]. Over the years, ^18^F-fluorodeoxyglucose (FDG)-positron emission tomography (PET) in combination with computed tomography (CT) has proved a valuable tool for detecting manifestations of lymphoma at diagnosis, and is nowadays considered state-of-the-art. On the basis of stage and other clinical risk indicators, Hodgkin lymphoma can be divided into three different risk-groups, these being early-favorable, early-unfavorable, and advanced disease. While the early stages commonly respond well to a combination of doxorubicin, bleomycin, vinblastine, and dacarbazine (ABVD), advanced Hodgkin lymphoma is more effectively treated with bleomycin, etoposide, doxorubicin, cyclophosphamide, vincristine, procarbazine, and prednisone (BEACOPP), for instance in its dose-escalated form (eBEACOPP) [3,4,5,6,7]. However, genotoxic treatment can lead to several complications and might be associated with increased long-term morbidity, including secondary malignancies and cardiovascular disease. A large survey among 1149 Hodgkin lymphoma survivors showed that optimal lymphoma control and primary cure are of utmost importance from the patient’s perspective [8]. In advanced stage, these goals can be most effectively achieved through an intensive chemotherapy regimen. However, as the group of patients with advanced Hodgkin lymphoma includes individuals with very different risk profiles, a substantial proportion of them, around 60% to 70%, would presumably be over-treated by eBEACOPP [9]. Biomarkers to assess the tumor under treatment are therefore of great importance for identifying those patients who respond very well to chemotherapy and in whom treatment intensity can be reduced without compromising the primary cure rate. On the other hand, individuals showing poor response might require escalation of therapy. A growing body of evidence strongly supports the use of FDG-PET for assessing changes in tumor metabolism during Hodgkin lymphoma treatment. As shown by large randomized trials, decisions regarding escalation [4] or de-escalation of therapy [3,7] can safely be based on PET results. The outstanding performance of FDG-PET in Hodgkin lymphoma most probably arises from its ratio of neoplastic to reactive microenvironment cells, which differs significantly from other lymphoma subtypes [10,11].

Diffuse large B-cell lymphoma (DLBCL) represents the most common subtype of non-Hodgkin lymphoma, accounting for 30% to 40% of all newly diagnosed cases [12]. Despite significant advances in the management of this malignancy, cure rates are still generally lower than for Hodgkin lymphoma, with approximately one-third of all individuals failing front-line therapy. Due to its superior sensitivity in the detection of nodal and extra-nodal lymphoma manifestations, FDG-PET is strongly recommended for staging patients with DLBCL [13]. Moreover, several international trials have examined whether PET imaging may be used early during treatment to separate individuals needing therapy intensification from good responders, that is, candidates for de-escalation [14,15,16,17,18]. As standard treatment, patients receive six chemotherapy cycles of cyclophosphamide, doxorubicin, vincristine, and prednisone alongside the monoclonal antibody rituximab (R-CHOP). Unlike early-stage disease, which can be considered for brief chemotherapy [19], advanced DLBCL with high risk, as defined by the International Prognostic Index, may require intensified treatment, including central nervous system prophylaxis [20,21]. The role of radiotherapy is still under discussion in DLBCL. Large randomized trials have indicated that subsequent consolidative irradiation of former bulky sites in advanced-stage patients may have a positive effect on outcome [22,23].

This article presents an overview of current PET applications in Hodgkin lymphoma and DLBCL. Furthermore, recent developments regarding imaging-directed therapy concepts for these individuals as well as future directions are discussed.

## 2. Initial Staging

Accurate and reproducible staging is crucial to determine the most appropriate treatment in both Hodgkin lymphoma and DLBCL. With high sensitivity for involved lymph nodes and extra-nodal disease, FDG-PET has become established as the standard of care for assessing all FDG-avid lymphomas, and is recommended by the 2014 Lugano criteria [24], which updated the established Ann Arbor classification [25].

### 2.1. Hodgkin Lymphoma

In the randomized multi-center U.K. RATHL trial [26], FDG-PET upstaged 14% of Hodgkin lymphoma patients, mainly due to bone marrow involvements, which had not been identified by biopsy. Downstaging occurred in 6% of cases, particularly as a result of splenomegaly with normal FDG uptake or enlarged but PET-negative lymph nodes. Several studies on Hodgkin lymphoma demonstrated that FDG-PET has a sensitivity even higher than biopsy for detecting bone marrow involvement. While bone marrow biopsy diagnoses about 5% of involvements, focal skeletal FDG lesions are seen in up to 20% of newly diagnosed patients [27,28,29,30,31]. The largest head-to-head comparison to date showed a sensitivity and negative predictive value (NPV) of 95% and 99.9%, respectively, for FDG-PET when taking positive biopsy as the reference standard [32]. Moreover, Pedersen et al. reported a significantly inferior progression-free survival (PFS) for individuals with focal bone lesions, independent of chemotherapy type [33]. Based on the high level of scientific evidence, there is a broad consensus that bone marrow biopsy can be abandoned in Hodgkin lymphoma patients staged by FDG-PET. Importantly, diffusely increased skeletal FDG uptake is rarely associated with positive biopsy results, and should therefore not be considered a sign of lymphoma [34].

In the vast majority of patients with Hodgkin lymphoma, PET-detected skeletal lesions do not influence therapy selection, as only < 1% of otherwise early-stage individuals were found to have bone marrow involvement on FDG-PET [32]. In a study of Picardi et al., PET-based staging at least resulted in an improved event-free survival compared to a historical cohort. However, an improved overall survival (OS) could not be shown by a randomized prospective trial [35].

### 2.2. DLBCL

**Figure 1:**
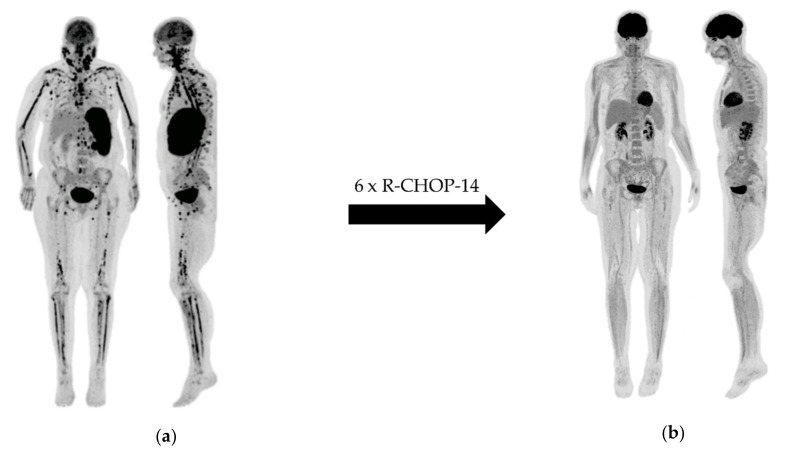
Female diffuse large B-cell lymphoma (DLBCL) patient with multifocal bone marrow involvement at baseline ^18^F-fluorodeoxyglucose (FDG)-positron emission tomography (PET) (**a**), which was missed by undirected bone marrow biopsy and remitted completely after six cycles of chemoimmunotherapy (**b**).

A retrospective analysis on DLBCL by Fuertes et al. reported upstaging in 15% of patients after FDG-PET [36]. Further studies focusing on subjects with DLBCL found similar results for non-Hodgkin lymphoma and rarely observed PET-based downstaging [37,38]. It should be taken into consideration that FDG-PET has proven to be less sensitive for identifying bone marrow involvements in aggressive non-Hodgkin lymphoma as compared to Hodgkin lymphoma [39,40]. On the basis of data from one Canadian and two Danish centers, Alzahrani et al. determined a sensitivity and NPV of 60% and 91%, respectively [41]. Similar findings emerged from a pooled analysis of the PETAL and OPTIMAL>60 study cohorts [42]. In this context, the limited sensitivity of PET can most likely be explained by the observation that positive bone marrow biopsy results of DLBCL patients are quite frequently associated with diffusely increased skeletal FDG uptake. On the other hand, the use of biopsy seems justified only when results could have a direct impact on treatment selection, for example in patients with limited-stage disease and lack of further risk factors. Importantly, bone marrow biopsy should be omitted in all individuals where involvement has already been proven with FDG-PET. Figure 1 shows a patient with multiple FDG-avid bone marrow lesions, which were missed by undirected biopsy.

## 3. Early Response Assessment

In 2009, the so-called Deauville score was introduced to meet the growing need for simple and reproducible PET interpretation in the setting of early response assessment [43]. Based on a visual comparison of lesional FDG uptake with that in the reference regions mediastinal blood pool and liver, it classifies residual tissue from 1 to 5 (Table 1). Several trials have shown superior accuracy and inter-observer agreement of PET-based lymphoma response assessment when using the Deauville criteria and contributed to a rapid integration of the proposed method into reporting routine. Importantly, graded assessment made interpretation more flexible, as this approach allows adjustment of the cut-off between positive and negative results depending on the clinical context.

### 3.1. Hodgkin Lymphoma

The key question of studies on early-stage Hodgkin lymphoma has been whether irradiation can be abandoned in the case of complete metabolic response after chemotherapy. In the U.K. RAPID trial [44], 602 stage IA and IIA patients without bulky mediastinal disease underwent PET imaging after three cycles of ABVD. Individuals with a negative PET scan, defined by Deauville scores 1 and 2, were randomly assigned to no further treatment or 30 Gy of involved-field irradiation. At a median follow-up of 60 months, patients receiving radiotherapy had a 3-year PFS of 94.6% compared to 90.8% in the non-irradiated group. The study did not prove non-inferiority of chemotherapy alone, as it was designed to exclude a difference in 3-year PFS of more than 7%. Thus, based on the data available thus far, we would not generally recommend omitting consolidative radiotherapy in PET-negative individuals after three courses of ABVD.

The H10 trial randomized 1950 patients with limited-stage favorable and unfavorable Hodgkin lymphoma to PET-guided or standard treatment with three to four cycles of ABVD plus involved-node radiotherapy [4]. In the experimental arm, PET scans were performed after two courses of chemotherapy (PET-2) and evaluated using the mediastinal blood pool as cut-off. Individuals with positive PET-2 received two cycles of eBEACOPP followed by involved-node radiotherapy, which resulted in a significantly improved 5-year PFS (90.6%) compared to standard treatment (77.4%). After a second randomization, PET-negative individuals either underwent combined-modality treatment or continued with ABVD alone. The latter arm had to be terminated after preplanned interim analysis due to a high number of relapses with the strategy omitting irradiation. Here again, the omission of radiotherapy in PET-negative patients appeared questionable.

Finally, the German Hodgkin Study Group (GHSG) conducted HD16 as a PET-driven trial for early-stage Hodgkin lymphoma [45]. In this study, patients either received standard combined-modality treatment with two courses of ABVD plus 20 Gy of involved-field radiotherapy or were assigned to the experimental arm where PET-negative individuals underwent no further treatment after chemotherapy. Any residual tissue showing an FDG uptake greater than the mediastinal blood pool was rated PET-positive. At a median follow-up of 45 months, PET-negative patients after combined-modality treatment had a 5-year PFS of 93.4% versus 86.1% with ABVD alone. Since PET-guided omission of irradiation resulted in worse disease control, the primary study objective was not achieved. However, among the 693 subjects receiving combined-modality treatment, PET-2 was predictive for therapy failure with a 5-year PFS of 93.2% in PET-negative patients versus 88.4% in the PET-positive group.

Based on the published data, radiotherapy should be generally recommended in limited-stage favorable Hodgkin lymphoma after treatment with two cycles of ABVD. Moreover, as revealed by the H10 trial, an intensification of therapy to eBEACOPP must be considered in patients with positive PET results. For intermediate-stage disease, the question for or against irradiation after two cycles of eBEACOPP and two courses ABVD has not yet been resolved. Here, we excitedly await the final results of the GHSG HD17 trial.

In advanced-stage Hodgkin lymphoma, several large studies strongly support the use of FDG-PET for tailoring treatment intensity. The RATHL trial included 1214 patients with stage IIB to IV or high-risk stage IIA disease and tested omission of bleomycin in ABVD cycles three to six after negative PET-2 using the liver as cut-off for PET interpretation [6]. Although results fell short of the prespecified non-inferiority margin, it should be noted that de-escalation of chemotherapy reduced the incidence of pulmonary side-effects without significant loss in efficacy. Individuals with Deauville scores 4 and 5 received either an accelerated BEACOPP version or eBEACOPP and had a 3-year PFS of 67.5%. Quite similar results emerged from the Italian phase III HD 0607 study, where treatment was escalated to BEACOPP in the case of positive PET-2. For these patients, the investigators reported a 3-year PFS of 57% [46]. The Lymphoma Study Association AHL2011 study examined de-escalation to ABVD in PET-negative patients after two cycles of BEACOPP and reported a 5-year PFS of 85.7% in the PET-driven arm versus 86.2% for continued treatment with BEACOPP (*p* = 0.65) [47]. Therefore, Casasnovas et al. concluded that FDG-PET after two cycles of induction BEACOPP can safely guide therapy in advanced-stage Hodgkin lymphoma and allows for the switching to ABVD in early responders without impairment of disease control.

Another de-escalation strategy was examined in the GHSG HD18 trial on advanced-stage Hodgkin lymphoma [7]. In patients receiving eBEACOPP from the beginning, negative PET-2 allowed shortening of treatment from six or eight to only four courses eBEACOPP, with a 5-year PFS of 90.8% and 92.2%, respectively. Importantly, severe infections and organ toxicities occurred significantly less often in subjects receiving four cycles of eBEACOPP. To avoid undertreatment, patients in the HD18 trial were randomized, rating a Deauville score of 3 as PET-positive. However, a further analysis revealed that only Deauville scores of 4 or higher signify a relevant risk regarding survival for individuals treated with eBEACOPP upfront, whereas Deauville scores of 1, 2, and 3 should be considered PET-negative [48].

### 3.2. DLBCL

In aggressive non-Hodgkin lymphoma, there are only few randomized studies investigating the role of interim PET as an early biomarker for treatment success or failure. The vast majority of trials on DLBCL have examined whether PET-positive patients benefit from therapy escalation. Depending on the timing of PET imaging, treatment was adapted after two to four therapy courses. However, differences in image evaluation and the absence of a control group made it difficult to draw definitive conclusions regarding potential benefits of PET-guided treatment for this lymphoma subset.

In their congress abstract on a study comprising 65 patients with early-stage DLBCL [49], Canadian researchers reported about the value of FDG-PET after three courses R-CHOP. Individuals with positive PET results received a fourth chemoimmunotherapy cycle instead of standard irradiation and were observed to have a superior 3-year PFS of 92% versus 60% when using radiotherapy alone. The German PETAL trial calculated the percentage change in maximum standardized uptake value (SUV_max_) between baseline and follow-up (ΔSUV_max_) for response stratification after two courses of R-CHOP and demonstrated semi-quantitative interpretation to be superior as compared with visual assessment [17,18]. Most of the 812 non-Hodgkin lymphoma patients recruited had DLBCL. All individuals with CD20-positive lymphoma and negative PET-2 were randomly assigned to four more cycles of R-CHOP either with or without two additional rituximab doses. Because the latter did not influence clinical outcome, therapy can be limited to six cycles of R-CHOP in the case of negative PET-2 without a loss of efficacy. In contrast, PET positivity was an independent biomarker for poor outcome that could not, however, be improved by treatment intensification.

Another prospective study on DLBCL, the PET-guided GAINED trial, compared obinutuzumab to rituximab plus chemotherapy in treatment-naïve patients younger than 60 years [50]. Individuals showing an early good response received the scheduled immunochemotherapy according to initial randomization, whereas slowly responding patients were treated with two courses of high-dose methotrexate followed by autologous stem-cell transplantation. Non-responders underwent salvage treatment according to local investigators. Based on the exploratory results of the LNH2007-3B study [51], PET positivity was defined as a decline of SUV_max_ in an FDG-avid target lesion of at least 66% or 70% from baseline to PET after 2 and 4 cycles of chemotherapy, respectively. This trial aimed to validate the ΔSUV_max_-driven consolidation in young patients with high-risk disease.

Finally, a recent analysis including 1977 DLBCL patients from different trials showed that FDG-PET is able to clearly discriminate between responding and non-responding individuals after two, three, or four chemotherapy cycles, while the optimal timing for identification of responders is after two courses [52]. Moreover, the authors recommended PET scanning after four cycles to identify poorly responding patients. Therefore, FDG-PET following chemotherapy courses two and four appears most suitable for future response-adapted trials and therapy regimens in aggressive non-Hodgkin lymphoma.

## 4. Late Response Evaluation

Before the era of FDG-PET, CT imaging was the standard modality for end-of-treatment stratification after chemotherapy. However, CT evaluation suffered from the inability of distinguishing between fibrotic masses and active residual lymphoma tissue, causing high rates of unconfirmed response [53]. Since the late 1990s, a number of studies have consistently demonstrated the high NPV of end-of-treatment PET and underlined its value for this application, particularly in Hodgkin lymphoma [54,55,56,57,58,59,60]. Spanish researchers examined 37 subjects with follicular lymphoma, as well as 72 Hodgkin lymphoma and 72 DLBCL patients, in order to compare the diagnostic performance of CT and FDG-PET/CT [13]. While CT was concordant with the defined reference methods in only 78% of cases, FDG-PET/CT showed an excellent agreement rate of 97.8%. In addition, PET imaging may be used during follow-up in certain situations. Here, it should be kept in mind that the value of both negative and positive results is limited. Even though FDG-PET might be able to detect relapses earlier than conventional imaging, it has been reported that only one- to two-thirds of positive scans actually indicate relapse and that that there is a significant number of false-negative results in this setting [60,61,62,63]. Moreover, the cost-effectiveness of routine PET scanning during follow-up could not be demonstrated [64]. This is why we recommend restricting the use of FDG-PET to selected cases, such as suspected relapse.

### 4.1. Hodgkin Lymphoma

In the GHSG HD15 trial on advanced-stage Hodgkin lymphoma, FDG-PET showed a NPV of 94.1% for tumor recurrence after BEACOPP chemotherapy [59]. The 4-year PFS of PET-negative patients who did not receive further treatment was 91.5% [65]. As a result of the HD15 study, radiotherapy is limited to PET-positive residual tissue in advanced-stage Hodgkin lymphoma after eBEACOPP therapy. Moreover, recently presented data have shown that consolidative irradiation might also be omitted in advanced-stage disease after treatment with ABVD [66]. Nevertheless, it is important to keep in mind that the positive predictive value of end-of-treatment PET is generally much lower than its NPV and that most PET-positive individuals stay disease-free after receiving additional radiotherapy, at least in early-stage Hodgkin lymphoma after ABVD and in advanced-stage disease treated with eBEACOPP.

### 4.2. DLBCL

Importantly, PET positivity after completion of R-CHOP therapy has to be interpreted as a sign of unfavorable prognosis in all stages of DLBCL. The prospective Swiss Group for Clinical Cancer Research 38/07 trial reported a significantly lower event-free survival of 48% in PET-positive patients versus 74% for those with negative FDG-PET after six courses of dose-dense R-CHOP (R-CHOP-14) [14]. In a population-based Canadian study, irradiation of PET-positive residual tissue after chemoimmunotherapy was shown to substantially improve clinical outcome so that the prognosis of subjects with positive and negative end-of-treatment PET were on a comparable level [15]. These observations could be confirmed by the interim analysis of the prospective German OPTIMAL>60 trial for patients with aggressive CD20-positive non-Hodgkin lymphoma [16]. Because this study had no randomization regarding FDG-PET, historic data from the very similar RICOVER-60 study are used for comparison. The latter found that individuals older than 60 years benefit additionally from irradiating former lymphoma bulk after six cycles of (R-)CHOP-14. According to the OPTIMAL>60 interim analysis, consolidating radiotherapy in the case of PET-positive bulky disease seems to have a positive effect on survival. Moreover, the Canadian and German data suggest that irradiation can be omitted in 42% to 60% of patients with PET-negative bulk.

## 5. Recent Advances and Future Directions

The Deauville score has been applied for therapy stratification in several clinical trials and evolved as an established method for monitoring response to treatment. However, there are ongoing discussions on how to interpret FDG uptake. Barrington et al. recently conducted a subsidiary analysis of the U.K. RAPID study for Hodgkin lymphoma patients and reported that a Deauville score of 5 was associated with an inferior outcome in their trial, where score 5 was defined quantitatively as three or more times the maximum liver uptake [67]. In the GHSG trials HD16 and HD18, Deauville score 3 proved not to be prognostic for progression or relapse, whereas Deauville score 4 did. Even though the majority of individuals with a Deauville score of 4 are cured, these patients show significantly impaired PFS and OS. Similar findings were reported for DLBCL [52]. This is why we recommend interpreting Deauville scores 4 and 5 as positive whenever cure is the primary aim.

**Figure 2:**
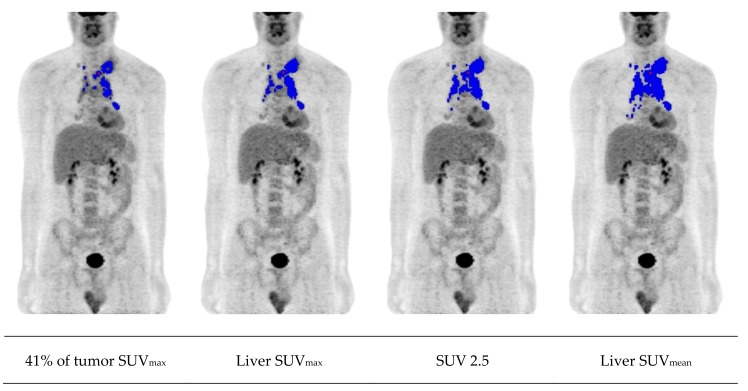
Metabolic tumor volume (MTV) measurement in a patient with advanced-stage Hodgkin lymphoma based on different thresholding methods. SUV, standardized uptake value; SUV_max_, maximum SUV; SUV_mean_, mean SUV.

Several working groups have recognized quantitative measures as an important tool in staging and response assessment, for instance SUV_max_ or ΔSUV_max_. Studies investigating the role of ΔSUV_max_ demonstrated its usefulness for response stratification in both Hodgkin lymphoma and DLBCL [68,69]. Additionally, FDG-PET provides biomarkers such as the metabolic tumor volume (MTV) or total lesion glycolysis (TLG), which incorporate information concerning tumor burden and disease activity. Cottereau et al. reported that the MTV improves baseline risk stratification of patients with early-stage Hodgkin lymphoma as compared to currently used staging systems [70]. Interestingly, individuals with early-stage unfavorable disease can even be subdivided into low- and high-risk categories based on the MTV and TLG, as shown elsewhere [71]. A retrospective analysis including 310 patients identified that pretreatment MTV is also a predictive factor for early response to eBEACOPP after two cycles of chemotherapy in advanced-stage Hodgkin lymphoma [72]. Moreover, metabolic measures were reported to have a prognostic value in non-Hodgkin lymphoma. Mikhaeel et al. demonstrated that MTV at staging is an important prognostic factor for DLBCL and that combining MTV with results of early PET response assessment improves the predictive power [73]. Similar findings emerged from a recently published analysis including 510 DLBCL patients treated within the PET-guided therapy optimization trial PETAL [74]. Interestingly, it has been also shown that MTV is a valid prognosticator in elderly individuals with DLBCL receiving R-CHOP. A study by Vercellino and colleagues found high pretreatment MTV to be significantly associated with inferior PFS and OS in this group [75]. Including patients from the LNH073B study, a French working group additionally examined the role of radiomic features characterizing lesion dissemination and reported that combining them with baseline MTV further improves risk stratification in DLBCL patients [76]. However, MTV calculation was carried out rather inconsistently in different working groups, which used both adaptive and fixed thresholds (Figure 2, Table 2). A recent review on the increasing importance of MTV addressed the need for standardization and pointed out that further analyses are needed to set common criteria [77]. Up until now, tumor delineation has been significantly more time-consuming in advanced-stage disease than in patients with fewer lesions. The application of machine learning and artificial intelligence can be expected to optimize complex workflows and will open up new perspectives in the future.

It needs to be kept in mind that the reliability of visual and quantitative response assessment can be impaired by inconsistent PET scanning protocols and image reconstruction methods [79]. Intravenous CT contrast media were found to affect PET images quantitatively due to an overestimation of attenuation factors in contrast-enhanced anatomic structure. This may have clinical consequences, particularly for response assessment, as the uptake values of lymphoma tissue rise less sharply than in the reference regions commonly used for PET interpretation [80]. Even though standardization is still lacking, quantitative FDG-PET has the potential to substantially improve prognostication in lymphoma. An integration of metabolic measures into clinical trials is currently planned by several working groups and will provide further evidence.

Over recent years, PET using FDG has brought many advances in the diagnosis and treatment of lymphoma patients, but there will most probably be a demand for more specific tracers other than FDG in future clinical practice. Indeed, the first successful trials with immune checkpoint inhibitors point to a major role for such substances in the first-line treatment of lymphomas [81]. *However, formerly established PET response criteria may fail in these novel therapy regimens due to false-positive findings caused by inflammatory changes. Therefore, the so-called RECIL scoring system, a combination of single-dimension measurement and PET criteria, was introduced for response evaluation by consensus of an international working group* [82]*. Not only new drugs but also the RECIL evaluation need prospective studies to confirm their clinical relevance.* In this context, it is worth mentioning that radiolabeled programmed cell death-ligand 1 (PD-L1) antibodies have recently proved to be useful for monitoring therapy-induced changes of PD-L1 expression in preclinical tumor models [83], and first clinical studies on solid malignancies have also demonstrated the feasibility of programmed cell death protein 1 (PD-1)/PD-L1 imaging [84]. Thus, highly specific tracers promise to improve our understanding of the dynamic tumor microenvironment and enable optimization of checkpoint inhibitor-based therapy strategies, not only in solid tumors but also in lymphoma patients. Imaging agents such as the chemokine receptor-targeting ^68^Ga-pentixafor [85] or the microenvironment-reflecting ^68^Ga-labeled fibroblast activation protein inhibitor (FAPI) [86] may offer further tools with which to elucidate the mechanisms of treatment and cure.

## 6. Conclusions

In FDG-avid lymphomas, PET has been established as the standard modality for both diagnosis and therapy monitoring (Table 3). PET-based staging is highly sensitive for detecting lymphoma lesions and therefore plays a crucial role in the context of contemporary treatment strategies. However, it should be noted that the positive predictive value of PET is rather moderate, with numerous non-malignant causes for FDG avidity. Importantly, undirected bone marrow biopsy can be safely omitted in Hodgkin lymphoma patients and individuals with DLBCL where involvement has already been proven by FDG-PET. As the overall predictive value of interim PET was found to be particularly high in Hodgkin lymphoma, it was established earlier in this disease than in other lymphoma subtypes. Personalized medicine is nowadays a reality, since escalation and de-escalation of chemo- and radiotherapy are adapted according to PET results. In aggressive non-Hodgkin lymphoma, baseline and interim PET imaging provide important information for disease prognosis as well. Moreover, end-of-treatment PET seems suitable to define whether a particular patient with DLBCL requires irradiation after chemotherapy or not.

Novel imaging-derived biomarkers such as the MTV or TLG may further individualize treatment of lymphomas. Hence, prospective trials are needed to validate the prognostic and predictive value of quantitative PET measures, given their great potential as guides for determining the future management of patients with lymphoma.

## Figures and Tables

**Table 1:** Deauville five-point scale for therapy stratification in patients with FDG-avid lymphomas.

Score	Criteria	Interpretation *
1	No FDG uptake	CR
2	FDG uptake lower than or equal to the mediastinal blood pool	
3	FDG uptake higher than the mediastinal blood pool but lower or equal to liver	
4	FDG uptake moderately increased compared to the liver	PR/SD/PD
5	FDG uptake markedly increased compared to the liver and/or new sites of disease	

* Response to treatment as defined by the Lugano classification [24]. CR, complete metabolic response; PR, partial metabolic response; SD, stable disease; PD, progressive disease.

**Table 2:** Common approaches for MTV calculation with their main characteristics.

Threshold	Advantages	Disadvantages
Fixed absolute (e.g., SUV 2.5 or 4.0)	High reproducibility	Overestimation if tumor lies adjacent to areas of high physiologic uptake
	Observer-independence	Underestimation in tumors that have many voxels with an uptake less than the threshold
Reference regions (e.g., liver or mediastinum) *	Adjusted to patient and scan	More time-consuming
		Low availability on commercial software
Fixed relative (e.g., 41% of tumor SUVmax)	Observer-independence	Overestimation in case of low lesion-to-background ratio
		Underestimation of tumors with heterogeneous uptake and high SUVmax
Adaptive (e.g., signal-to-background ratio)	Adjusted to patient and scan	More time-consuming
		Low availability on commercial software

* Thresholding method proposed by the PET Response Criteria in Solid Tumors (PERCIST) [78].

**Table 3:** Evidence-based recommendations on the use of FDG-PET before, during, and after treatment.

Indication	Hodgkin Lymphoma	DLBCL
Staging	+ + +	+ + +
Early response assessment	+ +	+ +
End-of-treatment	+ +	+ +
Follow-up	+/-	+/-

* + + +, standard modality; + +, standard—depending on therapy protocol; +/-, optional—recommended in selected cases, e.g., suspected relapse.
